# Microstructural evolution and nanograin coherence in VO_2_ thin films grown by pulsed laser deposition

**DOI:** 10.1038/s41598-026-52338-5

**Published:** 2026-05-19

**Authors:** Ayushi Rai, Vidar Hansen

**Affiliations:** Department of Mechanical and Structural Engineering and Materials Science, University of Stavanger, Stavanger, N-4036 Norway

**Keywords:** VO_2_ thin films, Pulsed laser deposition, Nanograin coherence, Grain morphology, Nanobeam diffraction, Confocal Raman spectroscopy, Materials science, Nanoscience and technology, Physics

## Abstract

Vanadium dioxide (VO_2_) thin films exhibit a reversible semiconductor–metal transition near room temperature, and their performance is strongly governed by microstructural and interfacial factors. In this study, VO_2_ thin films were deposited on Z-cut quartz substrates by pulsed laser deposition under varying oxygen partial pressures and substrate temperatures. Plan-view transmission electron microscopy (TEM) techniques such as selected-area electron diffraction (SAD) and nanobeam diffraction (NBD) as well as confocal Raman spectroscopy were employed to examine the microstructure and crystallographic features of the films. The VO_2_ films display a dense, polygonal morphology, composed of faceted grains with characteristic sizes of 50–200 nm. These polygonal grains are formed during deposition in the high-temperature metallic R phase and transform upon cooling into semi-coherent VO_2_ M1 nanograins with minimal lattice mismatch. The observed morphology is attributed to surface and interface energy anisotropy acting across the amorphous SiO_2_ interlayer, which promotes random in-plain orientation. Confocal Raman mapping further supports phase uniformity and orientation variation across the grains. Overall, this work provides new insight into the mechanisms governing polygonal grain formation and coherence within VO_2_ thin films on quartz, highlighting how interface-energy-driven growth can stabilize ordered microstructures even in the absence of epitaxial constraint.

**Supplementary Information:**

The online version contains supplementary material available at 10.1038/s41598-026-52338-5.

## Introduction

Vanadium dioxide (VO_2_) has been extensively studied owing to its reversible semiconductor-to-metal transition near room temperature, which makes it attractive for applications such as smart windows, sensors, and electronic devices.^1,2^ While the functional properties of VO_2_ are strongly influenced by its phase transformation behaviour, it is now well recognized that the underlying microstructure and crystallographic orientation of the thin films play an equally critical role in determining performance.^3,4^ For this reason, a detailed understanding of the growth and transformation mechanisms, grain morphology, and orientation relationships in VO_2_ thin films is essential.

In previous work, we have carried out cross-sectional studies on VO_2_ thin films deposited by pulsed laser deposition (PLD) for smart windows application.^5–9^ These studies have elucidated orientation relationships with substrates, interfacial layers, and strain gradients as a function of deposition parameters. In this work, plan-view transmission electron microscopy (TEM) was employed to examine the surface morphology and in-plane crystallographic orientations of the VO_2_ thin films. Unlike cross-sectional TEM, which requires complex specimen preparation and often exposes VO_2_ to prolonged ion milling, plan-view imaging allows direct observation of the as-grown surface with minimal mechanical or ion-induced damage.^10^ This approach is particularly advantageous for VO_2_, as the material is known to be sensitive to both electron beam exposure and localized heating.^11^ In addition to simplifying sample preparation, plan-view analysis provides complementary information on grain shape viewed from the growth direction, grain boundary configuration, and orientation relationships perpendicular to the growth direction that cannot be readily obtained from cross-sectional observations. Confocal Raman spectroscopy was further used for studying VO_2_ thin films, as it provides non-destructive, spatially resolved information on crystal phase and quality as well as strain state. The high lateral and depth resolution of the technique enables clear differentiation between monoclinic and rutile-type phase through their characteristic phonon frequencies.

The present paper focuses on VO_2_ thin films deposited on Z-cut quartz substrates by PLD. PLD offers precise control over stoichiometry and growth kinetics, enabling the fabrication of dense oxide films.^12^ The energetic ablation plume and tunable parameters, such as oxygen partial pressure and substrate temperature, allow systematic analyses of nucleation, grain growth, and phase formation in VO_2_ thin films.^13^ Despite extensive research on VO_2_ thin films, most studies have focused on epitaxial growth to the substrate or their functional properties, while the microstructural evolution of VO_2_ grown on quartz substrate with intrinsic amorphous layer remains largely unexplored. In particular, the origin and crystallographic basis of faceted, polygonal grain morphologies occasionally observed in non-epitaxial VO_2_ films remain poorly understood.

In this work, we investigate the microstructure of VO_2_ thin films deposited by pulsed laser deposition, emphasizing the origin of their faceted, polygonal morphology. By combining plan-view transmission electron microscopy (TEM), both systematic nanobeam diffraction (NBD) and selected-area diffraction (SAD) tilt-series, and confocal Raman spectroscopy, we reveal that these polygonal grains are composed of semi-coherent VO_2_ monoclinic M1 nanocrystallites that transform from the high-temperature tetragonal R phase. This study provides direct experimental evidence of semi-coherent grain assembly and interfacial decoupling in VO_2_ thin films on amorphous SiO_2_, offering new insight into morphology stabilization mechanisms that operate independently of epitaxial constraint.

## Materials and methods

VO_2_ thin films were deposited by pulsed laser deposition (PLD) using a KrF* excimer laser (COMPex PRO 205 F, Coherent; λ = 248 nm, τFWHM = 25 ns). The laser beam, operated at a pulse energy of 200 mJ and a repetition rate of 2 Hz, was focused onto a rotating metallic vanadium target (99.5%, Alfa Aesar) with a spot area of 10.2 mm^2^, corresponding to a fluence of ⁓2.0 J cm⁻^2^. The laser was incident on the target at an angle of 45° to ensure uniform ablation.

Quartz (0001) Z-cut substrates were positioned parallel to the target at a target–substrate distance of 50 mm. Prior to deposition, substrates were plasma cleaned using 1000 laser pulses under oxygen atmosphere to remove surface contaminants and improve film adhesion.

Film growth was carried out under varying oxygen partial pressures and substrate temperatures, while maintaining a constant number of 5000 deposition pulses. Oxygen pressures were varied between 0.8 and 1.6 Pa at a substrate temperature of 600 °C (Samples Q1–Q5), while Sample Q6 was deposited at 800 °C under 1 Pa O_2_ pressure to examine the effect of elevated growth temperature.

Following deposition, all films underwent in situ post-annealing for 1 h in the deposition chamber at the same oxygen pressure and substrate temperature as their respective growth conditions. A summary of the deposition parameters is provided in Table 1.

**Table 1:** Pulsed deposition conditions for sample Q1-6 with V (99.5%, Alfa Aesar) target and one hour of post deposition treatment at same pressure and temperature conditions.

Sample	Q1	Q2	Q3	Q4	Q5	Q6
Pressure O_2_ (Pa)	0.8	1	1.2	1.4	1.6	1
Temperature (°C)	600	600	600	600	600	800

### Transmission electron microscopy

The VO_2_ thin films were highly sensitive to electron-beam irradiation.^9^ To minimize beam damage and avoid complex sample preparation procedures, plan-view TEM specimens were prepared by mechanically scratching the films from the substrate.^10,14^ This approach enabled direct top-view observation of the film microstructure while preserving fragile grain features. The films were gently scratched using a silicon carbide (SiC) stylus and transferred onto lacey carbon–coated copper grids. Ultrasonication was deliberately avoided to prevent fragmentation or structural damage to the films.

Microstructural characterization was performed using a JEOL 2100 transmission electron microscope operated at an accelerating voltage of 200 kV. NBD was carried out using an electron probe size of approximately 1 nm to probe crystallographic orientations at the nanoscale. NBD patterns were acquired using a spot size 2 setting and a condenser aperture corresponding to α = 2. A double-tilt TEM holder was employed to perform systematic tilt-series diffraction experiments, and all diffraction data were collected with a 300 mm camera length.

Although the film thickness was not directly measured in this study, previous cross-sectional TEM investigations on VO_2_ films deposited under identical conditions with 5000 laser pulses indicate a thickness of ⁓100 nm. All TEM experiments were conducted at room temperature. Image analysis and distance measurements were performed using Radius software, while diffraction patterns were indexed using the Ideal software and JEMS.

## Confocal Raman spectroscopy

Confocal Raman measurements were performed using a LabRAM Soleil Raman spectrometer (HORIBA Scientific, Palaiseau, France) integrated with an OmegaScope scanning probe microscope. The confocal configuration enabled high spatial resolution and depth discrimination, allowing surface-sensitive structural analysis of the VO_2_ thin films.

A 532 nm excitation laser was employed in combination with an 1800 grooves mm^− 1^ diffraction grating. The objective lens had a numerical aperture (NA) of 0.9, providing a lateral optical resolution of ⁓360 nm. The resulting laser spot diameter at the sample surface was approximately 721 nm.

To prevent laser-induced heating and unintended phase transformation in VO_2_, the laser power was carefully limited to 0.23 mW. The acquisition time for each spectrum was optimized to 20 s to ensure adequate signal-to-noise ratio while minimizing thermal effects.

Two-dimensional spectral mapping was conducted over selected regions of the films to probe spatial variations in local structure and phase homogeneity. Raman maps were acquired with a lateral step size of 50 nm, enabling sub-micron resolution of microstructural features such as grain boundaries and orientation domains.

## Result and discussion

**Fig. 1:**
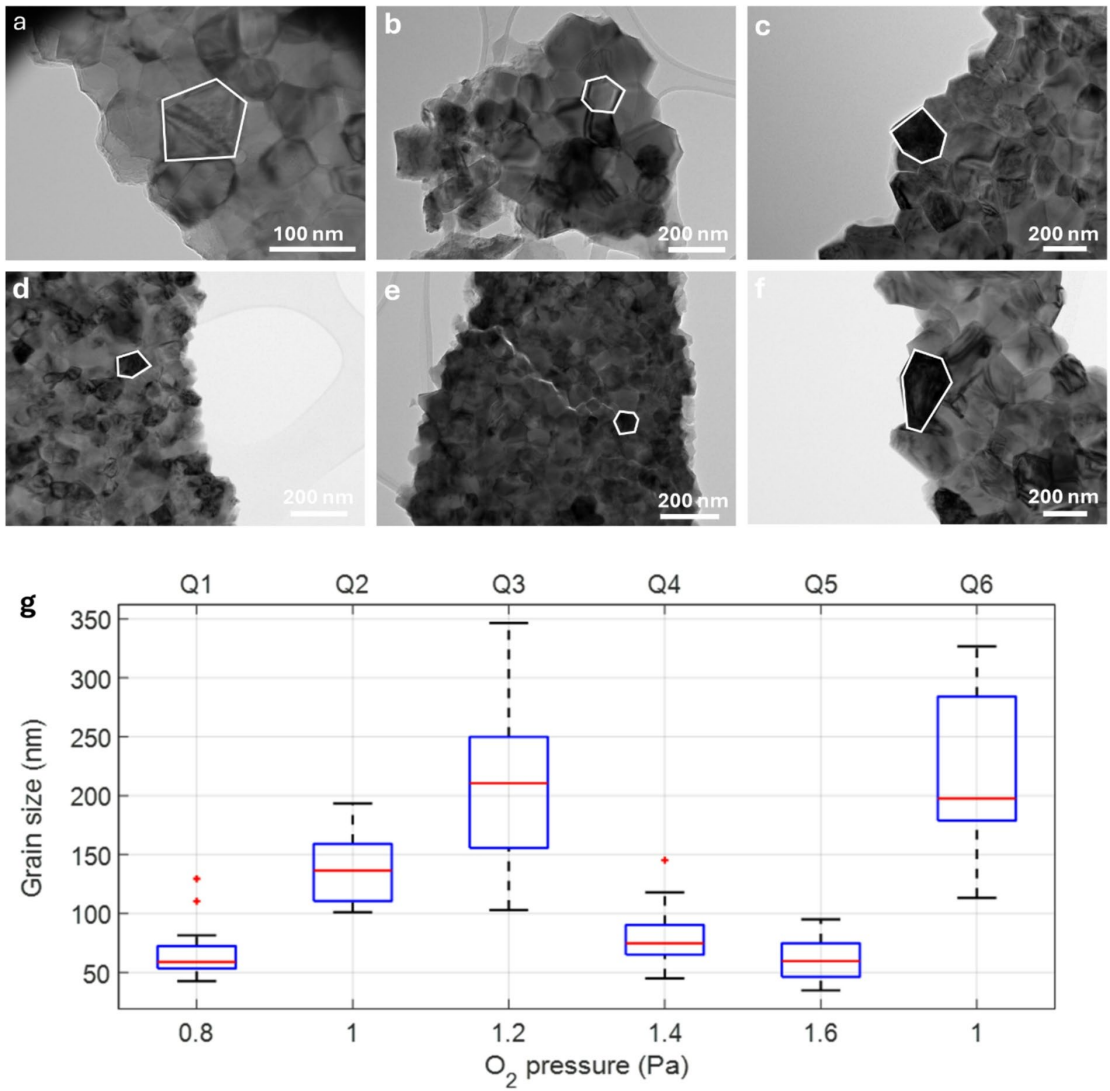
(**a**)-(**f**) Bright-field plan-view TEM images show the size distribution of polygonal grains from sample Q1 to Q6 respectively and one representative grain from each is outlined (**g**) Box plot of grain size distribution with deposition temperature 600 °C for sample Q1-Q5 and 800 °C for sample Q6.

Bright-field TEM images of the VO_2_ thin films revealed a dense polycrystalline microstructure composed of well-defined, faceted polygonal morphology as shown in Fig. 1a-f. These grains had sizes ranging from approximately 50 to 200 nm. While polygonal morphologies are known in hydrothermally synthesized VO_2_ particles, their occurrence in PLD thin films (especially on quartz) is not reported.^15^ A box plot (Fig. 1g) was used to illustrate the polygon grain size distribution for each sample. Here, the lower and upper end of the box represent 25th and 75th percentile respectively, whereas the red line denotes median value. The black dash denotes the minimum and maximum values along with the red cross signs which are for outliers. The outliers were chosen as more than 1.5 times the value of bottom and top of the box. The grain size was measured along the longest direction between parallel facets.

At first, with the increase in partial pressure, the grain size increased (Q1-Q3). When the temperature is kept constant and O_2_ partial pressure is increased, the grain size is expected to increase as reported previously by Bhardwaj et al.^16^ McGee et al., also reported that gas pressure is the most influential parameter for depositing VO_2_ M1 thin film.^17^ They also observed that with increasing temperature, grain size increase which can be observed for the sample Q6 where the deposition temperature was 800 °C as compared to all other samples being deposited at 600 °C. At elevated substrate temperatures, surface adatom mobility and diffusion length increase, enabling adatoms to migrate to energetically favourable sites. This extended diffusion length enables adatoms to reach existing islands or grain edges more readily, promoting coalescence and growth. At the same time, the increased atomic vibration reduces the likelihood of new nuclei forming, since adatoms are less frequently trapped near their landing sites. This results in a lower nucleation density and larger, well-faceted grains due to thermally enhanced surface diffusion and boundary migration.^18^ However, grain size dropped again above 1.2 Pa O_2_ pressure for Q4 and Q5 suggesting an optimal limit for grain size growth with respect to oxygen partial pressure. This is consistent with observations in other oxide thin film systems (e.g., Ga_2_O_3_, ZnO), where excessively high ambient pressure reduces mobility of the atoms by increasing plume scattering and decreasing kinetic energy. This limits surface diffusion, enhances nucleation density, and ultimately suppresses grain growth.^19,20^ The mechanical scratching of the films indicated poor film–substrate adhesion, likely due to the amorphous SiO_2_ intrinsic layer formation (observed in previous study^5^ and shown clearly in Supplementary Information Figure S1), yet strong internal cohesion among the VO_2_ polygonal grains.

**Fig. 2:**
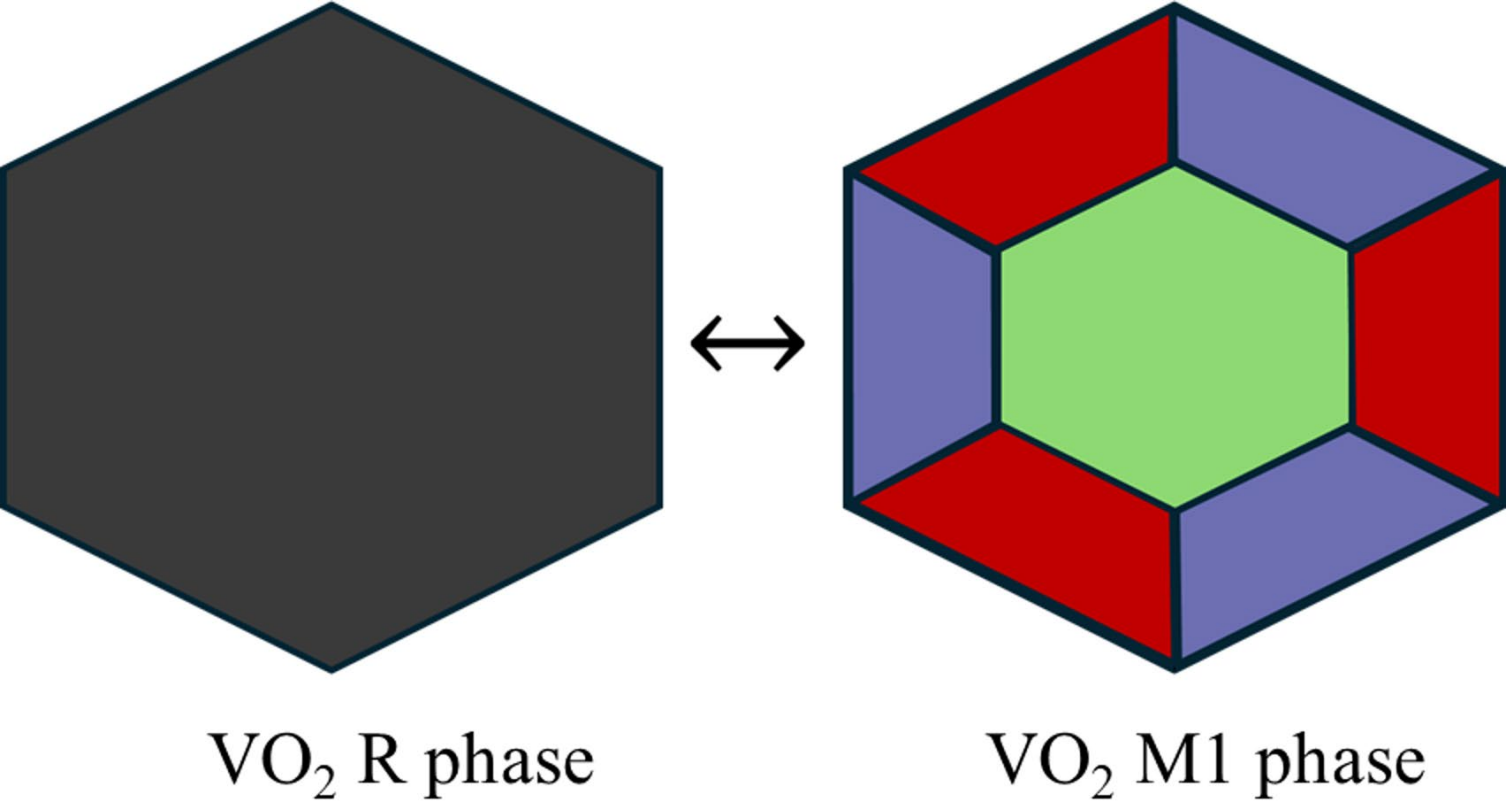
A schematic illustration of a polygon grain transformation from R phase to fragmented M1 phase where each colour of the fragment denotes one orientation.

These polygonal grains form during deposition in the high-temperature metallic VO_2_ R phase. Phase transformation from R to M1 phase results in fragmentation of the polygonal grain to several smaller M1 phase grains referred to as nanograin with a strict orientation relationship related to the mother R phase as illustrated in Fig. 2. This microstructural aspect is expected to influence local strain distribution and domain evolution during the VO_2_ phase transition, which are key factors governing switching behaviour and stability in large-area, non-epitaxial VO_2_ devices. It has been previously reported that with increasing O_2_ partial pressure, total resistance and metal to insulator transition (MIT) temperature increases and optical reflectance decreases.^21^ According to Jian et al., large grain size (low grain boundary density) contribute to excellent phase transition properties on glass substrates.^4^ Similarly, Brassard et al. observed that smaller grains had larger density of defects in the grain boundaries which lowered the resistivity in insulating phase and limited the conductivity in the metallic phase.^22^ Understanding and controlling this polygonal grain formation thus provides a pathway toward more predictable microstructural engineering of VO_2_ thin films on technologically relevant substrates such as quartz and glass.

**Fig. 3:**
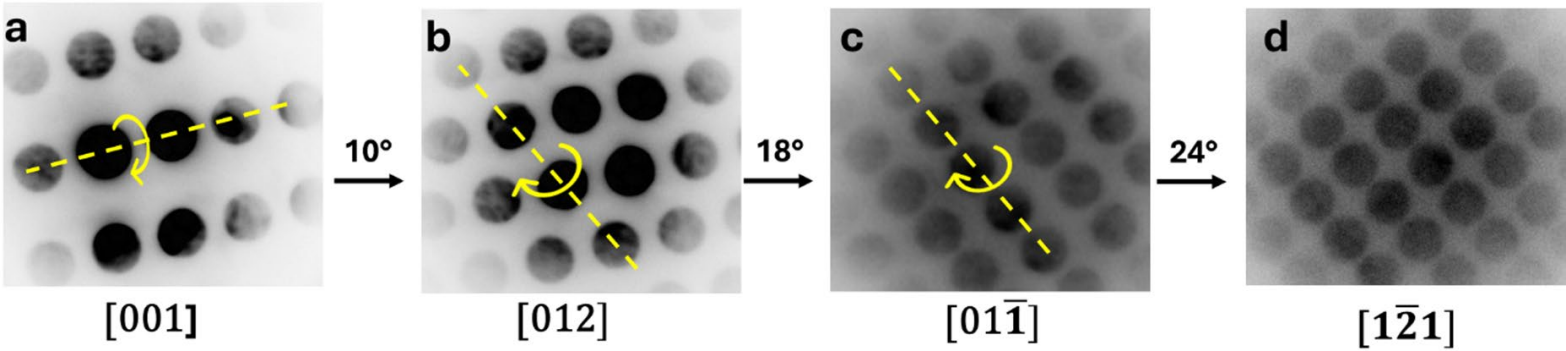
Systematic tilt series of NBD patterns from sample Q1 (**a**) [001] zone axis to (**b**) [012] zone axis after 10° tilt to (**c**) [01$\:\stackrel{-}{1}]$ zone axis with 18° tilt to (**d**) [1$\:\stackrel{-}{2}$1] zone axis after 24° tilt. Tilt direction is denoted by arrows while the dashed lines denote the axis of rotation.

To confirm the crystallographic phase of the VO_2_ thin films, systematic NBD tilt experiments along specific crystallographic rows were performed on samples Q1, Q2, and Q3. NBD was specifically employed because the polygonal grain sizes in these samples were too small to obtain SAD patterns from individual grains.

Phase identification from single NBD patterns proved challenging due to the presence of overlapping and coincident reflections that can be indexed to both the tetragonal VO_2_ R and monoclinic VO_2_ M1 structures (lattice parameters in Supplementary Information Table S1). In sample Q1, however, a well-defined tilt series was successfully acquired, as shown in Fig. 3a–d. The diffraction patterns obtained along this series could be indexed to both VO_2_ R and VO_2_ M1 phase when considered individually. For the VO_2_ R phase, the indexing from Fig. 3a–d corresponds to [11$\:\stackrel{-}{1}$], [$\:\stackrel{-}{2}$11], [1$\:\stackrel{-}{1}$1] and [1$\:\stackrel{-}{1}$0] whereas for the VO_2_ M1 phase the patterns can be indexed as [001], [012], [01$\:\stackrel{-}{1}]$ and [1$\:\stackrel{-}{2}$1].

To resolve this ambiguity, the relative tilt angles between successive zone axes were analysed. From Fig. 3a–d, the measured tilts between adjacent orientations along a specific crystallographic row were approximately 10°, 18°, and 24°, respectively. When these angular relationships are considered collectively, the crystallographic evolution of the diffraction projections follows the geometric constraints of the monoclinic M1 lattice rather than the tetragonal R phase.

Therefore, although individual diffraction patterns may be indexed to either structure, the full tilt-series analysis provides unambiguous confirmation that the observed polygonal domains correspond to the VO_2_ M1 phase.

**Fig. 4:**
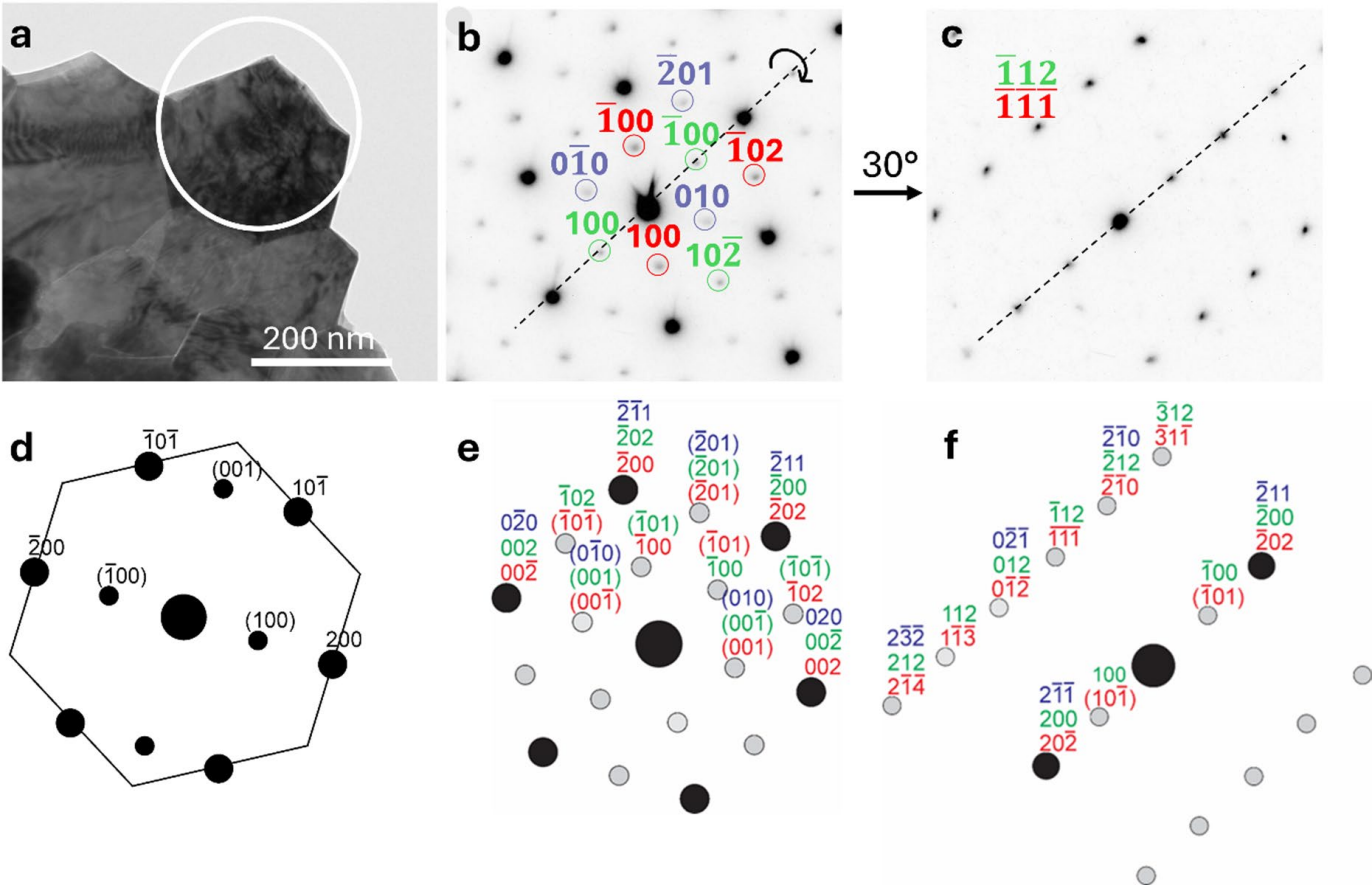
(**a**) Bright-field plan-view image of VO_2_ thin film in sample Q3 with the area where the diffraction pattern was collected from is marked by a white circle (**b**) Coinciding zone axes with colour-coded indexing where green denotes [010], red denotes [0$\:\stackrel{-}{1}0]$, and purple denotes [102] (**c**) SAD from sample Q3 acquired after a 30° tilt around an axis marked with dashed line in 4b (**d**)Schematic simulation of [010]_R_ illustrating the polygon structure formation (**e**-**f**) Corresponding schematic simulations of 4b and 4c used for detailed indexing of the diffraction patterns; reflections shown in brackets represent systematically absent spots due to extinction rules.

SAD patterns from plan-view analyses of sample Q3 (Fig. 4a) revealed a superimposition of the [010], [0$\:\stackrel{-}{1}0]$ and [102] zone axes indexed in Fig. 4b. A similar coexistence of multiple VO_2_ domains with [102], [010] and [0$\:\stackrel{-}{1}0]$ zone axes has been reported by Cheng et al.^23^ The orientation relationship between the M1 and R phases of VO_2_ is [010]_M1_║[100]_R_ and [102]_M1_║[010]_R_.^23^ In the tetragonal R phase, [100]_R_ and [010]_R_ are crystallographically equivalent, whereas in the monoclinic M1 phase, [010]_M1_ and [102]_M1_ are different.

Since the thin film deposition occurred at high temperature, VO_2_ initially grew in the R phase. Cheng et al. proposed that upon cooling through the phase transition, nanograins reorient into either < 010 > _M1_ or < 102 > _M1_ variants.^23^ In the present work, polygonal grain formation is promoted in the high-temperature R phase. Upon cooling, smaller nanograins (5–10 nm) form within these grains, adopting [010]_M1,_ [0$\:\stackrel{-}{1}0]$_M1_ and [102]_M1_ orientations.

## Coherence in VO_2_ thin films

To further investigate the coinciding SAD pattern shown in Fig. 4b, a tilt experiment was performed around the marked axis of rotation. After an approximate 30° goniometric tilt, an additional zone axis was observed, as shown in Fig. 4c. To verify whether three coinciding zone axes contribute to the SAD pattern in Fig. 4c, reciprocal space simulations were generated using Ideal Microscope software. When tilting 30° from the initial [010]_M1_ (Green), [0$\:\stackrel{-}{1}$0]_M1_ (red) and [102]_M1_ (purple) orientations around the same rotation axis, three corresponding zone axes were identified in the simulation.

This systematic tilt experiment was carried out to evaluate the degree of crystallographic coherence between the VO_2_ M1 nanograins. The experimental diffraction patterns overlap closely: strong reflections arise from contributions of all three orientations, whereas weaker reflections originate from one or two orientations. Based on the measured d-spacings (Supplementary Information Table S2), the lattice mismatch between these nanograins is calculated to be approximately 0.1–0.8% (Supplementary Information table S3), indicating semi-coherent interfaces within the original R-phase grain.

A detailed indexing of the SAD patterns was carried out with the aid of schematic simulations, as shown in Fig. 4d–f. Reflections indicated in brackets correspond to systematically absent spots arising from extinction rules and are therefore not physically present in the experimental patterns but are included for completeness of indexing. Figure 4d presents a schematic diffraction pattern corresponding to the [010]_R_ orientation. The projected lattice planes are highlighted to illustrate how the observed polygonal facets align with specific low-energy planes of the rutile VO_2_ phase, supporting the interpretation that facet formation during high-temperature growth is crystallographically guided.

Furthermore, Fig. 4f demonstrates that even after an accumulated tilt of approximately 30°, coherence is maintained between the three related orientations, [02$\:\stackrel{-}{1}$] (green), [1$\:\stackrel{-}{2}$1] (red), and [1$\:\stackrel{-}{2}$4] (purple). The preservation of crystallographic relationships across this tilt series confirms that the overlapping zone axes originate from a semi-coherent assembly rather than from randomly oriented grains.

The M1→R transition in VO_2_ is driven by the Gibbs energy difference (ΔG) between the two phases. In constrained thin films or nanograins, the total transformation energy also includes contributions from elastic strain and interfacial energy associated with maintaining lattice coherence across phase or grain boundaries.^24^ In the present system, the SAD patterns indicate that the [010]_M1_, [0$\:\stackrel{-}{1}$0]_M1_, and [102]_M1_ grains are crystallographically semi-coherent, resulting in minimal lattice mismatch at their interfaces. Consequently, both the elastic strain energy and the interfacial energy are reduced, lowering the overall energy barrier for the metal–insulator transition and facilitating the phase transformation.

## Polygonal grain formation

The honeycomb-like polygonal grains observed in samples Q2, Q3, and Q6 have not been explicitly reported for VO_2_ thin films on quartz substrate. Their formation can be understood by considering how the film nucleates and grows on the substrate and by considering the crystal-dependent surface energies of the VO_2_ R phase.

The SiO_2_ amorphous intrinsic layer present between the quartz substrate and the VO_2_ thin film has no crystal structure which removes any epitaxial constraint and allows, in principle, VO_2_ nuclei to grow with random in-plane orientations.^24^ SiO_2_ also provides a poor wetting surface, which promotes the formation of isolated three-dimensional islands during deposition rather than smooth, layer-by-layer growth. As these islands expand and grow together, grain boundaries form.

The appearance of faceted polygonal grains can be explained using Thompson’s model for grain growth in thin films.^24^ According to this model, when grains become as large as the film thickness, normal grain growth slows down. Further growth then becomes “abnormal,” meaning that certain grains grow at the expense of others because the energies of their boundaries and surfaces are not the same in all directions. Over time, these boundaries adjust to the lower-energy crystallographic planes. Grain boundaries that lie on high-energy planes tend to move quickly and disappear, while boundaries on low-energy planes grow slowly and remain. As a result, the surviving grains develop flat, well-defined facets that correspond to these low-energy planes.

For rutile VO_2_, theoretical studies have shown that certain crystal planes, especially the (110) (100) and (101) type planes possess lower surface energies than others.^25^ This observation is consistent with our diffraction results where the three coinciding zone axes, [010]_M1_, [0$\:\stackrel{-}{1}$0]_M1_, and [102]_M1_ correspond to [100]_R_ and [010]_R_ in high-temperature R. These orientations are precisely those associated with low-energy rutile planes, suggesting that, during the R-phase growth stage, grains preferentially developed facets aligned with these energetically favourable planes before transforming into the monoclinic M1 structure upon cooling.

Experimentally, VO_2_ films displaying preferential orientation of low-energy rutile planes—such as (110)_R_—have been reported to show flatter and well-faceted grains whereas films dominated by other orientations tend to display more irregular morphologies and increased roughness.^26,27^ In the present polycrystalline films, a distribution of grain orientations of R phase is therefore expected to contribute to local variations in surface topography. Consistent with this, our previous cross-sectional TEM studies revealed pronounced surface roughness in VO_2_ films grown on various substrates.^5,6^ The crystallographic orientation of individual grains, established during growth in the rutile phase, thus provides a mechanism for understanding the origin of surface roughness in these films. From an application perspective, this is particularly relevant for smart-window coatings, where increased surface roughness leads to enhanced visible-light scattering and reduced optical clarity.

### Confocal Raman spectroscopy analysis of VO_2_ thin films

**Fig. 5:**
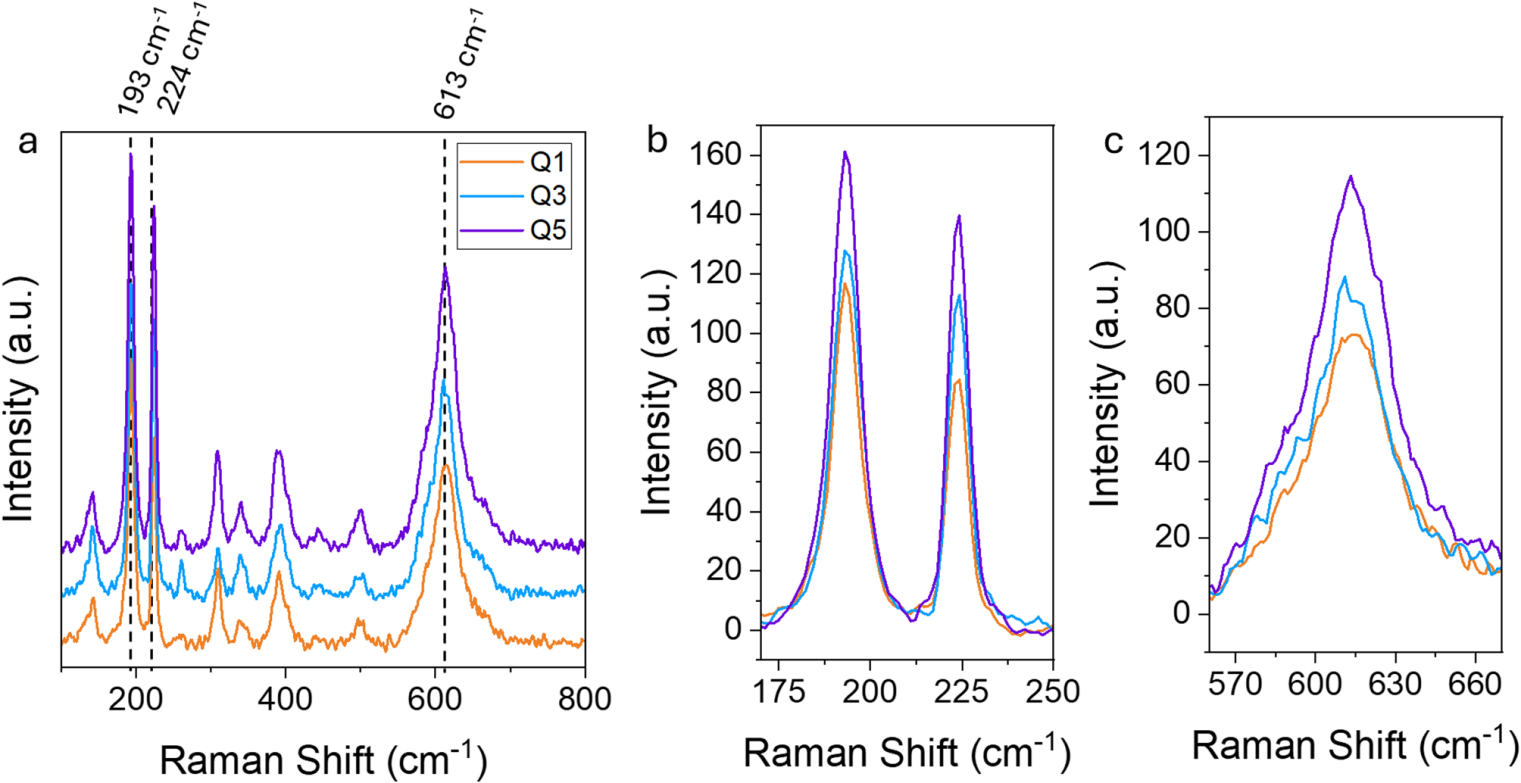
(**a**). Raman spectra of Q1, Q3 and Q5 stacked with an offset along Y axis (**b**) Raman peaks (193 cm^− 1^ and 224 cm^− 1^) contributing to V-V vibrational modes for comparison (**c**) Raman peak (613 cm^− 1^) contributing to V-O vibrational mode for comparison.

The Raman spectra of three representative samples (Q1, Q3, and Q5), corresponding to oxygen pressures of 0.8 Pa, 1.2 Pa, and 1.6 Pa, respectively, all deposited at 600 °C are presented in Fig. 5a. All spectra exhibit characteristic phonon modes of the monoclinic VO_2_ M1 phase at room temperature, including prominent peaks near 193 cm^− 1^, 224 cm^− 1^, 262 cm^− 1^, 308 cm^− 1^, 340 cm^− 1^, 391 cm^− 1^, 442 cm^− 1^, 500 cm^− 1^, and a broad feature near 613 cm^− 1^, consistent with earlier reports on VO_2_ M1.^9,28^ These peaks are attributed to various V─ O lattice vibrations and V─V dimer-related modes specific to the M1 phase. A detailed peak assignment was carried out in our earlier work^9^ and close-up of the relevant peaks has been shown in Figs. 5 and c.

**Table 4:** FWHM and peak intensity comparison of samples Q1, Q3 and Q5.

Sample	FWHM for peak position		Peak intensity	
	193 cm^− 1^	613 cm^− 1^	193 cm^− 1^	613 cm^− 1^
Q1	55.32	151.40	116	73
Q3	46.18	125.16	126	88
Q5	36.94	96.87	160	114

A systematic evolution of the Raman features with oxygen partial pressure is observed. As summarized in Table 4, the full width at half maximum (FWHM) of both the 193 cm^− 1^ and 613 cm^− 1^ peaks decreases from Q1 to Q5, while their intensities increase. This indicates a progressive improvement in crystalline quality, phase purity, and lattice ordering with increasing oxygen pressure. Sample Q1, deposited at 0.8 Pa, shows broader peaks and weaker intensity, suggesting higher defect density and partial disorder. In contrast, sample Q5 (1.6 Pa) exhibits sharp and well-defined phonon modes, indicative of improved stoichiometry and reduced defect concentration.

Post deposition annealing was carried out on all the samples at their respective O_2_ partial pressure. Annealing is known to improve crystalline quality and increase average grain size improving phase purity.^29,30^ Annealing in the oxygen environment also promotes homogenization by oxygen diffusion and consequently Raman peak sharpening.^31^ At higher oxygen partial pressure (1.6 Pa), oxygen vacancies and possibility of other detrimental phase formation decreases, leading to improved lattice order and sharper phonon modes despite the reduction in polygonal grain size.^32,33^.

Although smaller grain sizes generally lead to an increased grain-boundary area and therefore a higher density of structural defects, grain size is not the sole factor governing defect concentration in VO_2_ thin films. In the present study, oxygen stoichiometry plays a dominant role. The higher oxygen partial pressure used for sample Q5 suppresses the formation of oxygen vacancies and improves the overall stoichiometric balance of the film. As a result, despite its smaller grain size, Q5 exhibits reduced point-defect concentration associated with oxygen deficiency.

### Local structural variations revealed by confocal Raman spectroscopy

**Fig. 6:**
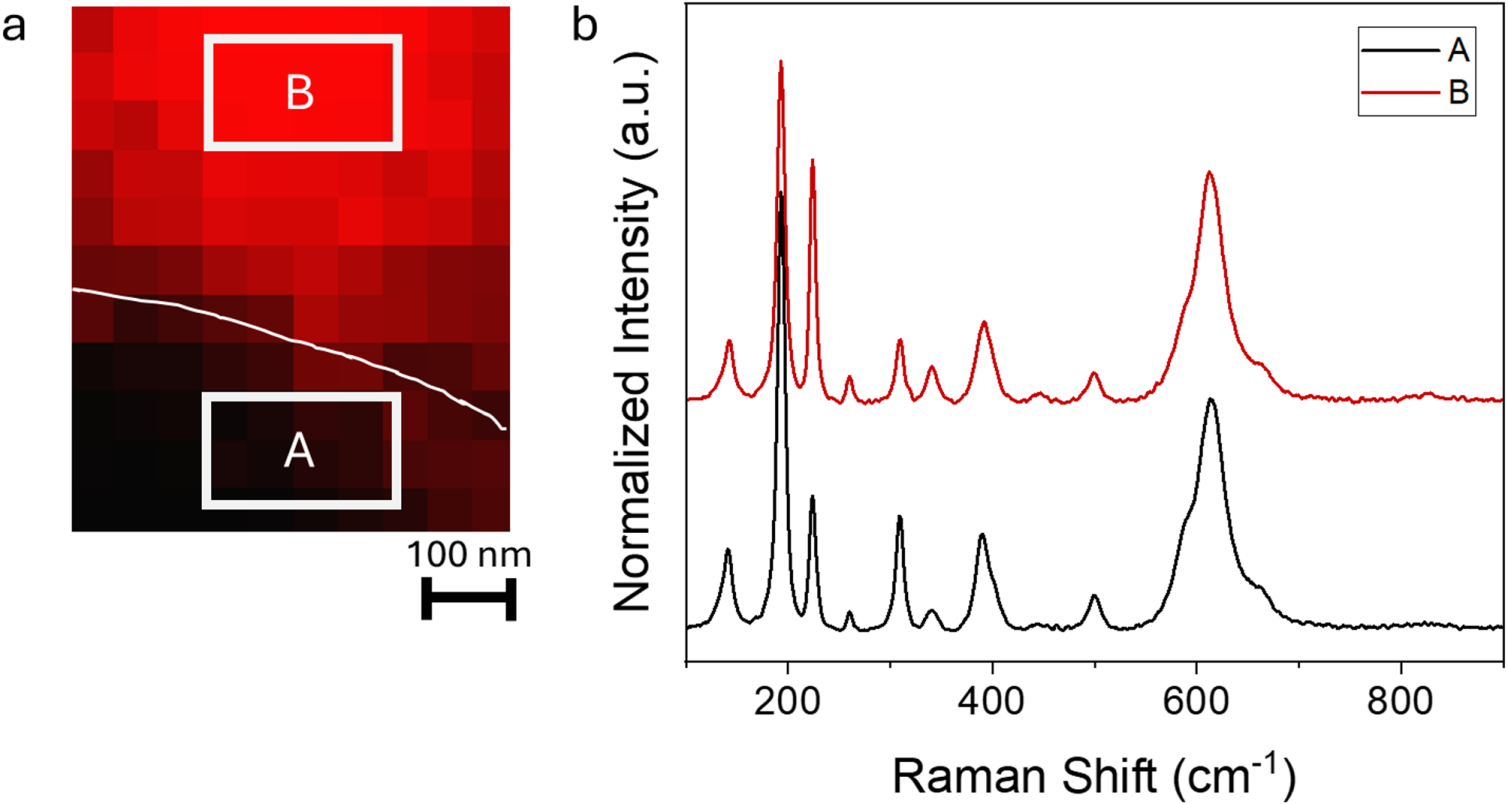
(**a**). Confocal Raman map showing the interface between to different orientations of VO_2_ M1 grains in sample Q3, constructed from Raman signal at 224 cm^− 1^ with a step size of 50 nm (**b**) Raman spectra from region A and B marked in 5a, normalized with the intensity of the peak at 613 cm^− 1^.

To probe spatial variations in local structure, two-dimensional confocal Raman mapping was performed on the VO_2_ thin films. Figure 6a shows a Raman intensity map of sample Q3 constructed from the 224 cm^− 1^ mode with a lateral step size of 50 nm. A region exhibiting pronounced structural inhomogeneity was identified as shown in the map 6a, and two distinct areas, labelled A and B, were selected from it for further analysis.

The averaged Raman spectra from these two regions are shown in Fig. 6b, normalized with respect to the 613 cm^− 1^ peak intensity. Both regions displayed the characteristic phonon signatures of the monoclinic M1 phase, with no evidence of any detrimental phases, confirming that the observed contrast does not arise from phase mixing. Instead, a significant variation was observed in the intensity ratio of the 193 cm^− 1^ and 224 cm^− 1^ peaks.

Polarization Raman studies on VO_2_ have previously reported that the phonon mode for 193 cm^− 1^ and 224 cm^− 1^ corresponds to V─V vibrations in different directions.^34^ Strelchuk et al. reports that phonon mode for 193 cm^− 1^ corresponds to V─V vibrations along c_M1_ axis. The phonon mode for 224 cm^− 1^ corresponds to V─V vibrations along a_M1_ axis.^35^ Therefore, the variation in the intensity ratio between these two modes directly reflects differences in crystallographic orientation. Given the high lateral resolution of confocal Raman and the polygonal grain size observed by TEM investigation, the contrast in Fig. 6a strongly suggests the presence of a grain boundary.

Although electrical measurements were not performed within the scope of this study, the observed dependence of microstructure and stoichiometry on oxygen partial pressure has important implications for the functional properties of VO_2_. Oxygen vacancies act as donor-like defects that increase the free carrier concentration, thereby reducing the resistivity of the insulating phase and diminishing the sharpness of the metal–insulator transition. This behaviour is consistent with reports on comparable PLD-grown films, where samples deposited at lower oxygen partial pressures exhibited reduced resistance contrast and broadened hysteresis, while films grown at higher oxygen pressures demonstrated improved switching characteristics.^8^.

Beyond electrical transport, these structural variations are also relevant for optical performance in smart-window applications. Higher substrate temperature, as in sample Q6, resulted in increased grain size, consistent with the observations of Barimah et al. Larger grains generally reduce grain boundary density and can promote denser film formation, thereby minimizing porosity-related surface defects.^36^ Surface morphology further influences optical clarity: increased roughness enhances diffuse scattering of visible light and reduces transparency, whereas well-faceted and structurally coherent grains may help preserve visible transmittance while maintaining infrared modulation.

Together, these considerations suggest that precise control of oxygen stoichiometry, grain growth, and nanograin coherence is critical not only for structural stability but also for optimizing the electronic and optical performance of VO_2_ thin films.

## Conclusion

This study provides new insight into how microstructural evolution and interfacial effects govern the morphology and crystallographic semi-coherence of VO_2_ thin films grown on quartz Z-cut substrates by pulsed laser deposition. The emergence of faceted, honeycomb-like polygonal grains, perpendicular to the growing direction, demonstrates that, even in the absence of epitaxial constraints, the interplay between surface and interface energies can drive highly textured polygonal growth. Beyond the polygonal morphology from the precursor R phase, plan-view TEM and diffraction analyses reveal that these grains are composed of semi-coherent VO_2_ M1 nanograins with strict orientation relationship and minimal lattice mismatch to each other, a structural feature that has not been explicitly demonstrated for non-epitaxial VO_2_ films on quartz substrates. These observations suggest that interfacial energy minimization and partial crystallographic coherence play a central role in stabilizing the polygonal grain network during the R→M1 phase transformation with minimal atomic rearrangement between the atoms in the reversible transformation kinetics.

This study focuses primarily on microstructural evolution and coherence in VO_2_ films and does not include electrical or optical measurements of the metal–insulator transition. The conclusions are drawn from plan-view TEM and Raman data rather than cross-sectional TEM or in situ growth analysis. Nevertheless, the consistent correlation between morphology, crystallographic semi-coherence, and local structural variations provides a robust basis for understanding polygonal grain formation in VO_2_ thin films grown on quartz substrate.

From a broader perspective, this structural framework provides a route toward microstructural engineering of VO_2_ thin films, where grain coherence and interfacial strain can be tailored to influence phase-transition behaviour and device stability in large-area, non-epitaxial systems.

## Supplementary Information

Below is the link to the electronic supplementary material.

Supplementary Material 1

## Data Availability

The datasets generated and analysed during the current study are available within the article and its Supplementary Information. Additional data, including raw TEM and Raman data, are available from the corresponding author upon reasonable request.

## References

[1] Granqvist, C. G. Recent progress in thermochromics and electrochromics: A brief survey. *Thin Solid Films*. **614**, 90–96. 10.1016/j.tsf.2016.02.029 (2016).

[2] Granqvist, C. G. Smart windows and intelligent glass façades. *Smart Materials Bulletin* 9–10 (2002). (2002). 10.1016/S1471-3918(02)80152-6

[3] Jian, J., Chen, A., Chen, Y., Zhang, X. & Wang, H. Roles of strain and domain boundaries on the phase transition stability of VO_2_ thin films. *Appl. Phys. Lett.* **111**, 153102. 10.1063/1.4991882 (2017).

[4] Jian, J., Chen, A. P., Zhang, W. R. & Wang, H. Y. Sharp semiconductor-to-metal transition of VO_2_ thin films on glass substrates. *J. Appl. Phys.* **114**10.1063/1.4851655 (2013).

[5] Rai, A. et al. *in Analytical and Experimental Methods in Mechanical and Civil Engineering* 3–12 (Springer Nature Switzerland, 2024).

[6] Rai, A. et al. Impact of nanostructural features of thermochromic VO_2_/TiO_2_ bilayers on their electrical and optical properties. *Appl. Surf. Sci.* **720**, 165367. 10.1016/j.apsusc.2025.165367 (2026).

[7] Rai, A. et al. Microstructural Investigations of VO_2_ Thermochromic Thin Films Grown by Pulsed Laser Deposition for Smart Windows Applications. *Inorganics* **10**, 220. (2022).

[8] Kuncser, V. et al. in *in Analytical and Experimental Methods in Mechanical and Civil Engineering*. 57–65 (eds Pavlou, D.) (Springer Nature Switzerland, 2024).

[9] Rai, A., Bienz, S., Hansen, V. F., Zenobi, R. & Kumar, N. Nanoscale Structural Insights into Thermochromic VO_2_ Thin Films Using Tip-Enhanced Raman Spectroscopy. *ACS Appl. Mater. Interfaces*. **17**, 32625–32634. 10.1021/acsami.5c04827 (2025). 40404575 10.1021/acsami.5c04827 PMC12147077

[10] Mayer, J., Giannuzzi, L. A., Kamino, T. & Michael, J. T. E. M. Sample Preparation and FIB-Induced Damage. *MRS Bull.* **32**, 400–407. 10.1557/mrs2007.63 (2007).

[11] Zhang, Y. et al. Artificially controlled nanoscale chemical reduction in VO_2_ through electron beam illumination. *Nat. Commun.* **14**, 4012. 10.1038/s41467-023-39812-8 (2023). 37419923 10.1038/s41467-023-39812-8 PMC10329014

[12] McGee, R. et al. Sharpness and intensity modulation of the metal-insulator transition in ultrathin VO_2_ films by interfacial structure manipulation. *Phys. Rev. Mater.* **2**, 034605. 10.1103/PhysRevMaterials.2.034605 (2018).

[13] Shepelin, N. A. et al. A practical guide to pulsed laser deposition. *Chem. Soc. Rev.* **52**, 2294–2321. 10.1039/d2cs00938b (2023). 36916771 10.1039/d2cs00938b PMC10068590

[14] Shen, Y. Q. et al. in. *IEEE 24th International Symposium on the Physical and Failure Analysis of Integrated Circuits (IPFA)* 1–4 (2017). (2017).

[15] Xu, H. Y., Xu, K. W., Ma, F. & Chu, P. K. Hexagonal VO_2_ particles: synthesis, mechanism and thermochromic properties. *RSC Adv.* **8**, 10064–10071. 10.1039/C8RA00716K (2018). 35540861 10.1039/c8ra00716k PMC9078730

[16] Bhardwaj, D., Goswami, A. & Umarji, A. M. Synthesis of phase pure vanadium dioxide (VO_2_) thin film by reactive pulsed laser deposition. *J. Appl. Phys.* **124**, 135301. 10.1063/1.5046455 (2018).

[17] McGee, R. et al. Effect of process parameters on phase stability and metal-insulator transition of vanadium dioxide (VO_2_) thin films by pulsed laser deposition. *Acta Mater.* **137**, 12–21. 10.1016/j.actamat.2017.07.025 (2017).

[18] Kumi-Barimah, E., Anagnostou, D. E. & Jose, G. Phase changeable vanadium dioxide (VO_2_) thin films grown from vanadium pentoxide (V_2_O_5_) using femtosecond pulsed laser deposition. *AIP Adv.* **10**, 065225. 10.1063/5.0010157 (2020).

[19] Liao, Y. et al. Effect of deposition pressure on the structural and optical properties of Ga_2_O_3_ films obtained by thermal post-crystallization. *CrystEngComm* **20**, 133–139. 10.1039/C7CE01567D (2018).

[20] Muchuweni, E., Sathiaraj, T. S. & Nyakotyo, H. Effect of O_2_/Ar flow ratio on Ga and Al co-doped ZnO thin films by rf sputtering for optoelectronic device fabrication. *Mater. Res. Bull.* **95**, 123–128. 10.1016/j.materresbull.2017.07.029 (2017).

[21] Chiu, T. W., Tonooka, K. & Kikuchi, N. Influence of oxygen pressure on the structural, electrical and optical properties of VO_2_ thin films deposited on ZnO/glass substrates by pulsed laser deposition. *Thin Solid Films*. **518**, 7441–7444. 10.1016/j.tsf.2010.05.019 (2010).

[22] Brassard, D., Fourmaux, S., Jean-Jacques, M. & Kieffer, J. C. El Khakani, M. A. Grain size effect on the semiconductor-metal phase transition characteristics of magnetron-sputtered VO_2_ thin films. *Appl. Phys. Lett.* **87**, 051910. 10.1063/1.2001139 (2005).

[23] Cheng, S. et al. Inherent stochasticity during insulator–metal transition in VO_2_. *Proceedings of the National Academy of Sciences* 118, e2105895118 (2021). 10.1073/pnas.2105895118 10.1073/pnas.2105895118 PMC8449351 34493666

[24] Thompson, C. V. Grain Growth in Thin Films. *Annu. Rev. Mater. Sci.* **20**, 245–268. 10.1146/annurev.ms.20.080190.001333 (1990).

[25] Mellan, T. A. & Grau-Crespo, R. Density functional theory study of rutile VO_2_ surfaces. *J. Chem. Phys.* **137**, 154706. 10.1063/1.4758319 (2012). 23083183 10.1063/1.4758319

[26] Genchi, S. et al. Growth of vanadium dioxide thin films on hexagonal boron nitride flakes as transferrable substrates. *Sci. Rep.* **9**, 2857. 10.1038/s41598-019-39091-8 (2019). 30814545 10.1038/s41598-019-39091-8 PMC6393539

[27] Yu, J. H., Nam, S. H., Lee, J. W. & Boo, J. H. Enhanced Visible Transmittance of Thermochromic VO₂ Thin Films by SiO₂ Passivation Layer and Their Optical Characterization. *Mater. (Basel)*. 9. 10.3390/ma9070556 (2016). 10.3390/ma9070556 PMC5456844 28773679

[28] Shvets, P., Dikaya, O., Maksimova, K. & Goikhman, A. A review of Raman spectroscopy of vanadium oxides. *J. Raman Spectrosc.* **50**, 1226–1244. 10.1002/jrs.5616 (2019).

[29] Dou, Y. K. et al. Oxidizing annealing effects on VO_2_ films with different microstructures. *Appl. Surf. Sci.* **345**, 232–237. 10.1016/j.apsusc.2015.03.044 (2015).

[30] Liang, J. et al. Enhancement of metal-insulator transition performance of VO_2_ thin films by conventional furnace annealing. *Thin Solid Films*. **730**, 138709. 10.1016/j.tsf.2021.138709 (2021).

[31] Koussi, E. K. et al. Synthesis of vanadium oxides by pulsed laser deposition and rapid thermal annealing. *Appl. Surf. Sci.* **521**, 146267. 10.1016/j.apsusc.2020.146267 (2020).

[32] Lee, S., Meyer, T. L., Park, S., Egami, T. & Lee, H. N. Growth control of the oxidation state in vanadium oxide thin films. *Appl. Phys. Lett.* **105**10.1063/1.4903348 (2014).

[33] Kamat, A. et al. Tunable insulator–metal transition in epitaxial VO_2_ thin films via strain and defect engineering. *Nanoscale Adv.* **6**, 5625–5635. 10.1039/D4NA00682H (2024). 39296281 10.1039/d4na00682h PMC11406615

[34] Basu, R. et al. Polarized Tip-Enhanced Raman Spectroscopy in Understanding Metal-to-Insulator and Structural Phase Transition in VO_2_. *J. Phys. Chem. C*. **123**, 11189–11196. 10.1021/acs.jpcc.8b12401 (2019).

[35] Strelchuk, V. V. et al. Effect of structural disorder on the modification of V–V and V–O bond lengths at the metal-dielectric phase transition in VO_2_ thin films. *Mater. Sci. Semicond. Process.* **174**, 108224. 10.1016/j.mssp.2024.108224 (2024).

[36] Barimah, E. K., Boontan, A., Steenson, D. P. & Jose, G. Infrared optical properties modulation of VO2 thin film fabricated by ultrafast pulsed laser deposition for thermochromic smart window applications. *Sci. Rep.* **12**, 11421. 10.1038/s41598-022-15439-5 (2022). 35794203 10.1038/s41598-022-15439-5 PMC9259692

