# Supplementary material for: Microstructural evolution and nanograin coherence in VO2 thin films grown by pulsed laser deposition
Source: Sci Rep. 2026 May 19;16:20334. doi: 10.1038/s41598-026-52338-5 (PMC13328520; doi:10.1038/s41598-026-52338-5)
Supplement: Supplementary file 1 — Supplementary Material 1 [file 41598_2026_52338_MOESM1_ESM.docx]

**Supplementary information**

**Microstructural Evolution and Nanograin Coherence in VO_2_ Thin Films Grown by Pulsed Laser Deposition**

Ayushi Rai^1^* and Vidar Hansen^1^

1. Department of Mechanical and Structural Engineering and Materials Science, University of Stavanger, N-4036 Stavanger, Norway

*Corresponding author email: [ayushi.rai@uis.no](mailto:ayushi.rai@uis.no)

Figure S1 TEM images showing SiO_2_ amorphous layer

Table S1 Lattice parameters of VO_2_ M1 and VO_2_ R phase

Table S2 hkl and d-values table for M1 phase.

Table S3 Strain calculated between relevant hkl values


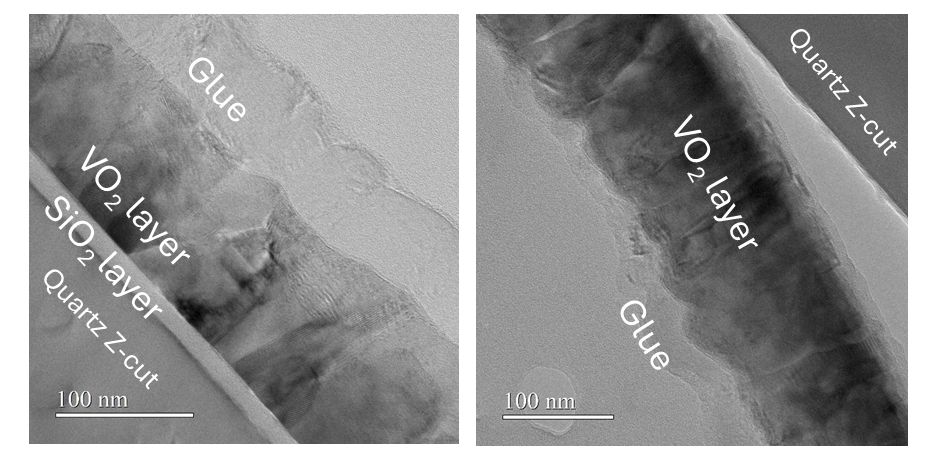


Figure S1(a): Bright field TEM images aquired from similar samples on Quartz substrate showing SiO_2_ amorphous layer formation. (b) and poor adhesion to the substrate.

Table S1. Lattice parameters of VO_2_ M1 and VO_2_ R phase

| Material | Space group | Lattice Parameters (Å) | | | | Density g/cm^3^ |
| --- | --- | --- | --- | --- | --- | --- |
|  |  | a | b | c | β (°) |  |
| VO_2_ M1 | P21/c (14) | 5.75 | 4.53 | 5.35 | 122.66 | 4.6 |
| VO_2_ R | P42/mnm (136) | 4.55 |  | 2.86 |  | 4.6 |

Table S2. Relevant hkl and corresponding calculated d-values of M1 phase used in this work where reflections assumed to be symmetrically equivaled are hkl, h-kl, -h-k-l and -hk-l.

| **hkl** | **d (Å)** |  | **hkl** | **d (Å)** |
| --- | --- | --- | --- | --- |
| **1 0 -1** | **4.86** |  | **0 0 2** | **2.26** |
| **1 0 0** | **4.84** |  | **0 2 0** | **2.26** |
| **0 0 1** | **4.53** |  | **2 1 -2** | **2.14** |
| **0 1 0** | **4.52** |  | **2 1 0** | **2.13** |
| **1 1 -1** | **3.31** |  | **1 2 -1** | **2.05** |
| **1 1 0** | **3.30** |  | **1 2 0** | **2.05** |
| **0 1 1** | **3.20** |  | **0 1 2** | **2.02** |
| **2 0 -1** | **2.87** |  | **0 2 1** | **2.02** |
| **1 0 -2** | **2.68** |  | **3 0 -2** | **1.88** |
| **1 0 1** | **2.67** |  | **3 0 -1** | **1.87** |
| **2 0 -2** | **2.43** |  | **2 0 -3** | **1.78** |
| **2 1 -1** | **2.42** |  | **2 2 -1** | **1.77** |
| **2 0 0** | **2.42** |  | **2 0 1** | **1.77** |
| **1 1 -2** | **2.30** |  | **3 1 -2** | **1.73** |
| **1 1 1** | **2.29** |  | **1 0 -3** | **1.73** |

Table S3. Absolute strain calculated from hkl values indexed in Figure 4.

| **(hkl)_1_** | **(hkl)_2_** | **Absolute strain (%)** |
| --- | --- | --- |
| **20-2** | **-2-11** | **0.41** |
| **10-1** | **100** | **0.41** |
| **10-2** | **101** | **0.75** |
| **010** | **001** | **0.44** |
| **020** | **002** | **0.44** |
| **21-2** | **210** | **0.46** |
| **31-2** | **31-1** | **0.17** |
| **11-2** | **111** | **0.43** |
| **021** | **012** | **0.13** |
| **11-3** | **112** | **0.29** |
